# Start-Up’s Road to Disruptive Innovation in the Digital Era: The Interplay Between Dynamic Capabilities and Business Model Innovation

**DOI:** 10.3389/fpsyg.2022.925277

**Published:** 2022-06-21

**Authors:** Ke Zhang, Lijie Feng, Jinfeng Wang, Guo Qin, Huailiang Li

**Affiliations:** ^1^School of Management Engineering, Zhengzhou University, Zhengzhou, China; ^2^China Institute of FTZ Supply Chain, Shanghai Maritime University, Shanghai, China; ^3^Henan Judicial Police Vocational College, Zhengzhou, China

**Keywords:** disruptive innovation, start-up, digital technology, dynamic capability, business model innovation

## Abstract

The emergence and infusion of digital technologies bring greater chances for start-ups to conduct disruptive innovation through digital entrepreneurship. Despite the existed business practices, the happening mechanism of start-up’s disruptive innovation in the digital economy context remains unclear. This study aims to understand the evolutionary mechanism and fulfillment path start-ups’ disruptive innovation in the digital era. The longitudinal case study is conducted for a Chinese Internet start-up that successfully launched disruptive innovation under the digital economy background. Adopting a process perspective, this study analyzes the evolutionary phases of digital disruptive innovation. Moreover, this study identifies the digital technologies adoption, dynamic capabilities deployment, and business model innovation as the key pillars, and their interactions. Finally, this study induces and proposes its evolution mechanism and fulfillment path models. This study enriches the research scope of disruptive innovation and digital entrepreneurship. This study can offer theoretical guidance for the start-ups’ disruptive innovation in the digital era, and practical implications for implementing a digital catching-up strategy.

## Introduction

The emergence and diffusion of digital technologies have driven the business, economy, and society to enter a digital era (Cai et al., 2022). Digital technologies are playing a crucial role in stimulating business creativity, and extending organizational boundaries (Curzi et al., 2019; Trenerry et al., 2021). Numerous new opportunities for entrepreneurial activity are created through digitalization. Accordingly, fundamental transformations and even disruptions in many industries are triggered and accelerated by ubiquitous and versatile digital technologies (Trischler and Li-Ying, 2022). Digitalization is becoming a societal necessity, as well as a challenge (Zhang et al., 2022). Both threats and opportunities are, respectively, brought to established companies and newcomers from the lens of disruptive innovation (Roblek et al., 2021). Especially for the agile but resource-constraint start-ups, they are usually considered with obvious disadvantages in traditional competition mode. The digital era has provided lower entry barriers and greater opportunities to implement entrepreneurial activities and achieve disruptive innovation (Vial, 2019). Hence, investigating the start-up disruptive innovation process in the digital era is a valuable topic.

This topic is drawn from several related literature streams where the research gaps are identified. The disruptive innovation literature has matured in its definition clarification (Martínez-Vergara and Valls-Pasola, 2020) and influence factors analysis (Zach et al., 2020). And breakthroughs can be created by continuous technological innovation (Wang et al., 2022), product or service innovation (Zheng et al., 2021), or business model innovation (Schmidt and Scaringella, 2020). Moreover, it has been highlighted that disruptive innovation is not an event but a process (Snihur et al., 2018). In parallel, the digital transformation of innovation and entrepreneurship research is advancing rapidly. It also describes digital transformation as a process (Vial, 2019) and emphasizes the disruptive characteristic of digital technologies (Lyytinen and Rose, 2003). Despite their convergence in holding a process view, and both connections with disruption, there have been limited studies concerning the disruptive innovation issue in the digital era.

Moreover, few studies have focused on the established large enterprises (Fraser and Ansari, 2021), while considered less on start-ups. Despite the existed business practices, academic attentions have fallen behind to pay enough attentions to related theoretical development. As significant digital innovation and entrepreneurship forces, the start-ups with great disruptive innovation potential should not be neglected (Herrmann et al., 2018), especially for their growth mechanism. Considering the different features of start-ups and established firms, the findings from the established firms may not be suitable for start-ups. There still exists a black box about the realization process of start-up’s digital disruptive innovation, including its triggering conditions, key actions, and expected results. The absent understandings of the dynamic evolutionary mechanism and path of start-up’s digital disruptive innovation will hinder its success rate and increase failure risks.

As for the key activities supporting the disruptive innovation or digital innovation and entrepreneurship process, existing studies have provided various insights such as business model innovation (Trischler and Li-Ying, 2022), agile development (Ghezzi and Cavallo, 2020), innovation ecosystem (Chen et al., 2021), organizational capability building (Li et al., 2018). In particular, dynamic capabilities are considered one of the promising theoretical foundations for understanding the firm’s digital entrepreneurship in the volatile, uncertain, complex, and ambiguous business world (Vial, 2019). In addition, business model innovation involving the change of value architecture is regarded as the primary means to achieve disruptive innovation (Silva and Grützmann, 2022) and digital transformation (Vaska et al., 2021). Previous studies have discussed them separately without considering their interactions. The employment of a single perspective may lead to a deficiency in explaining the complex fulfillment mechanism of disruptive innovation in the digital context.

According to the literature synthesis, three research gaps can be identified. Firstly, the research has kept silent on the disruptive innovation occurring in the emerging digital economy context, especially for the start-up firms. Recent studies have also appealed for embedding the disruptive innovation theory into the emergent contexts (Si and Chen, 2020) to extend its application boundaries. The second gap exist in adopting the process view to map the happening mechanism of start-up’s digital disruptive innovation. Its evolutionary process and fulfillment path remain unclear. Thirdly, the important roles of dynamic capabilities and business model innovation have been recognized in disruptive innovation or digital innovation and entrepreneurship process. However, their links have not obtained enough attention which may provide fine-grained illustrations for opening the mechanism black box. Current research provides little understanding of how the integration of digital technology adoption, dynamic capability deployment, and business model innovation might trigger and push the start-ups’ disruptive innovation. Hence, this study proposes the following research questions: What is the fulfillment path of start-up disruptive innovation in the digital era, and how does it evolve?

To answer the questions above, this study conducts an exploratory single case study for a Chinese digital start-up, ByteDance. ByteDance has realized disruptive innovation as a start-up and changed the competitive landscape of the Internet industry. Based on the case analysis results, this study has proposed the evolution mechanism model and fulfillment path model of start-up disruptive innovation in the digital era. The contributions of this study can be summarized into three aspects. Firstly, this study explores the evolutionary phases of start-up’s disruptive innovation in the digital context from a process view. Secondly, this study identifies the key pillars underpinning the fulfillment path as digital technology, dynamic capabilities, and business model innovation. Finally, this study concerns on the interactions between the dynamic capabilities, and business model innovation to illustrate its evolution and fulfillment mechanism. This study has contributed to enhancing the communications of the related literature streams. It also hopes to offer practical references for start-ups’ disruptive innovation and digital entrepreneurship.

The rest of the study is organized as follows. Section “Literature Review” reviews the previous studies from three major literature streams. Section “Research Design” introduces the contents of the research design. Section “Findings” reveals the analysis findings of the case company. Section “Discussion” proposes the conceptual models of start-up’s disruptive innovation in the digital era based on the case findings and discusses the evolution mechanisms and fulfillment path in detail. Finally, the conclusion section summarizes the theoretical and practical implications and limitations.

## Literature Review

### Disruptive Innovation and Digital Entrepreneurship

Disruptive innovation was firstly proposed to describe the incumbents’ loss of dominant position due to the new entrants’ innovative technologies, products, services, or business models (Christensen and Bower, 1996; Christensen, 1997; Christensen and Raynor, 2003). Scholars have devoted much effort to enrich the connotations of disruptive innovation from various perspectives. The first stream focused on the different types of innovation activities to fulfill disruptive innovation. Particularly, they mainly involve disruptive technology innovation (Chen et al., 2020), disruptive product or service innovation (Govindarajan et al., 2011), and disruptive business model innovation (Roblek et al., 2021). The second stream adopts the process perspective to understand disruptive innovation. It refers to a progressive process in which an innovator originates in the low-end or new market that is usually neglected and gradually moves from the fringe to the mainstream position with the continuous product or service improvements.

The third one is developed based on the outcome orientation. This stream usually defines the goal of disruptive innovation as the displacement of traditional incumbents or the sharing of the market by changing the established development trajectory (Zhang et al., 2019). The multi-dimension analysis above has revealed the typical characteristics of disruptive innovation, based on which Si and Chen (2020) have proposed a renewed definition for it. Disruptive innovation is a process in which firms initially target the low-end or new market to provide these non-mainstream customers with inferior but attractive technologies, products, or services and gradually penetrate the mainstream market through dynamic improvements, which can change the competitive landscape or even replace the incumbents. This study follows this definition which serves as the theoretical basis for subsequent analysis.

In parallel with the debate on disruptive innovation, another research stream, digital innovation, and entrepreneurship, has been rapidly developed due to the emergence of digital technologies. Various insights have been contributed, involving digital business models (Klos et al., 2021), digital entrepreneurship process (Lin and Maruping, 2022), platform strategies (Jääskeläinen et al., 2021), and digital ecosystem (Beltagui et al., 2020), etc. Among them, the disruptive implications of digital technologies for businesses have been greatly highlighted (Nambisan et al., 2019). Then, several studies have noticed the linkages between digital transformation and disruptive innovation (Fraser and Ansari, 2021; Zhang and Zhu, 2021). However, scant attention has paid to disruptive innovation’s evolution mechanism and fulfillment path in the emerging digital economy context. Moreover, they have mainly concerned the incumbent firms or small and medium enterprises, considering less the star-ups which are the important innovation forces in the digital era.

### Dynamic Capabilities

Dynamic capability was early defined as the ability of a company to integrate, build, and reconfigure internal and external capabilities to respond to a rapidly changing environment (Teece et al., 1997). It concerns the creation and development of an enterprise’s sustainable competitive advantages. Scholars have enriched the understandings of dynamic capability from various perspectives, including the strategy integration perspective, resource integration process perspective (Eisenhardt and Martin, 2000), and organizational learning perspective (Zollo and Winter, 2002).

In the increasingly turbulent business environment, dynamic capability is widely employed as a more useful theoretical perspective to understand the firm-level (Hopp et al., 2018), even national-level (Hameed et al., 2021) innovation development. Especially in the disruptive innovation field, several studies have tried to explore the roles of dynamic capability. For example, Wang et al. (2020) uncovered the fulfillment of disruptive innovations could be reached through exploratory, exploitative, and transformative learning capacities, which focused on the aspect of absorption capability. Furthermore, Schmidt and Scaringella (2020) examined the relationship between dynamic capabilities and disruptive business model innovation, mediated by value proposition innovation. These discussions showed the merits of combining the dynamic capabilities and disruptive innovation literature based on the evidence from different industries without considering the digital context.

With the advent of the digital economy, novel management phenomena have further driven the infusion of the dynamic capability view. Ilmudeen (2021) proposed the IT-enabled Dynamic Capabilities (ITDCs), including sensing, coordinating, learning, integrating, and reconfiguring, which can facilitate the firm’s strategic agility and innovative capability. Chen and Lin (2021) built the Sense-Transform-Drive (STD) conceptual model based on the dynamic capabilities theory to clarify business intelligence’s core capabilities. Moreover, Guo et al. (2021) utilized the dynamic capabilities perspective to explain the digital start-up’s alignments between business model innovation and technological innovation. These studies can provide a theoretical basis for this study by linking the potential of dynamic capability with the highly uncertain digital era. This study followed Teece (2007) and classified the dynamic capabilities from three dimensions, sensing, seizing, and reconfiguring capability.

### Business Model Innovation

Business model is the architecture that describes the mechanisms for an enterprise to create, deliver, and capture value (Teece, 2010). In the architecture paradigm, a business model is considered to consist of a series of interrelated elements where various insights have been drawn (Osterwalder and Pigneur, 2010; Zott et al., 2011). Among them, wide agreements can be obtained by identifying the core components of business model into three dimensions, value proposition, value creation, and value capture (Howell et al., 2018). Meanwhile, business model innovation also receives great attention, with related research focusing on the business model content design (Zhou et al., 2021) and its evolutionary innovation process (Costa Climent and Haftor, 2021). By following the logic of business model components, business model innovation involves a new combination of components and the architectural relationships of connected components (Foss and Saebi, 2017; Budler et al., 2021).

The existing studies have highlighted the crucial role of business model for disruptive innovation (Benzidia et al., 2021) and considered business model innovation as the important path or part of disruptive innovation (Cozzolino et al., 2018; Alberti-Alhtaybat et al., 2019). Furthermore, related research has explored the disruptive business model design framework (Sundström et al., 2020) or considered the combination with the emerging context such as sharing economy (Si et al., 2021). The application of digital technology is extending the scope of business model deployment and accelerating business model innovation (Lu and Yu, 2022). A series of novel business models embedded in the digital context have been developed, such as the platform business model (Jääskeläinen et al., 2021), which reveal the novel characteristics of servitization, agility, and value co-creation. Moreover, the dynamic evolutionary feature of business model in the rapidly developing digital era appears more obvious (Bohnsack et al., 2021).

In addition, the relationships between business model innovation and dynamic capabilities also attracted increasing academic interests. Several recent studies have highlighted dynamic capabilities’ supporting and enabling roles for business model innovation (Velu, 2017; Heider et al., 2021; Santa-Maria et al., 2021). Meanwhile, another study (Randhawa et al., 2021) uncovered the co-evolution of dynamic capabilities and business model innovation to align with each other. Teece (2018) demonstrated the interdependent relationships among business models, dynamic capabilities, and strategy theoretically and appealed further empirical studies for them. These insights are beneficial for advancing the understandings of various types of innovation mechanisms in depth.

### Literature Summary

By synthesizing the literature segmentations above, this study identified the following gaps. Firstly, the existing studies have discussed the issue of disruptive innovation and digital entrepreneurship, respectively. However, the research has kept silent on the overlapping of the dual contexts, i.e., the disruptive innovation phenomenon in the digital context, especially its happening mechanism. Secondly, the dynamic capabilities and business model innovation have been, respectively, highlighted as key activities for fulfilling disruptive innovation or digital transformation. However, scant research has further explored the roles of their interactions in such a strategic change process, despite the recent findings of their connections. Therefore, this study considers disruptive innovation in the digital era as a strategic change process and explores the happening and evolution mechanisms from the perspective of dynamic capabilities, business model innovation, and their interactions.

## Research Design

### Research Method and Case Selection

This study employs the case study method for the following reasons. Firstly, the chief object of this study is to explore how the start-up’s disruptive innovation fulfill and evolve in the digital context. To answer the “How” questions, it is suitable to adopt the exploratory and longitudinal single case research method to mine the case vertically and deeply (Yin, 2013). Then, dynamic evolution process of the research phenomena over time can be unfolded and the fulfillment path can be summarized. Secondly, the single case analysis is conducive to constructing a causal evidence chain and unveiling the fulfilling mechanism of disruptive innovation by identifying the activities and interactions of business model innovation and dynamic capabilities at different periods. Finally, considering the existing literature gaps on this topic, the observations and conclusions based on the exemplary case can help deepen understandings of similar events (Gioia et al., 2013).

This study chose Beijing ByteDance Technology Co., Ltd. (ByteDance for short) as the sample case with the following three aspects of reasons. Firstly, this case is consistent with the defined research object of start-up and specific digital context. ByteDance entered the mobile Internet industry as a start-up and has launched its products and services by deeply applying digital technologies. Secondly, the case company’s growth history is a relatively complete evolutionary process of disruptive innovation. Despite as a start-up founded in 2012, ByteDance has grown into one of the leading firms in the digital media field and exceeded many large or incumbent Internet enterprises. It thus can be regarded as the prominent representative of start-up disruptive innovation in the digital era. The evolutionary mechanisms and details of disruptive innovation can be fully explored. Finally, there are ample data reflecting the case company’s change process to guarantee the research reliability and validity. The author team has kept close contact with the sample case based on the industry-university-research cooperation, providing sufficient case data and information.

### Data Collection

This study collected the case data mainly from semi-structured interviews, second-hand information, and participatory observation. It cohered with the principle of triangulation verification to reduce the data source biases. Firstly, over 20 interviewees participated in the semi-structured interviews, including senior managers, distinct business units’ managers, technicians, and front-line employees. The duration of all interviews was kept at 0.5 to 2 h. The obtained information was recorded and transformed into text within 24 h. Due to the COVID-19 pandemic, the interviews were held discontinuously in offline or online ways.

Secondly, internal documents and public documents of the case company were collected as the second-hand information. The former included the corporate annual reports, technical manuals, statistical data, publicity materials, etc. The latter involved the published papers, official websites, authoritative media’s news reports on the case company, their patent information, etc. Thirdly, data also came from the authors’ long-term observation and experience notes. The author team visited the local office of the case company several times and learned about their digital tools, business model change, and corporate culture. One author has deeply participated in the case company’s media platform as the user and creator, to accumulate intuitive perceptions.

### Data Analysis

The study employed the grounded theory building approach to conduct the bottom-up induction analysis for the collected case data (Corbin and Strauss, 1990). The iterations between the data and theory were carried out through three recursive procedures: open coding, axial coding, and selective coding (Eisenhardt, 1989; Eisenhardt and Graebner, 2007). This study firstly established a database by integrating the multi-source data. Then, a chronology was formed to cover all significant events concerning the development of the case company’s business model, dynamic capability, digital technology, and the achieved results.

The three coding and analysis phases above are detailed as follows. (1) The open coding involves the labeling, conceptualization, and categorization of the original data. The authors coded the case independently, and the coding results then were adapted and agreed upon through regular discussions. (2) The axial coding focuses on the potential logical relationships among the obtained initial categories. This study referenced the coding paradigm, “condition-action/interaction-result,” proposed by Strauss and Corbin (1998) to cluster the initial categories into fewer main categories. (3) The selective coding aims to obtain the core categories by further abstracting the main categories based on their connotation and nature. Furthermore, by examining the logical connections between the categories at different levels, a theoretical framework can be inducted and deduced until it achieves theoretical saturation.

## Findings

The findings of case analysis will be detailed in this section. This study starts from introducing the background of the case company. ByteDance was founded in 2012, later than many other internet giants in China. However, it is one of the first companies to apply artificial intelligence (AI) to the mobile Internet scene. Over the past decade, it has owned over a dozen of apps and one billion registered users. In 2020, ByteDance was rated as the most valuable unicorn by CB Insights^1^ and listed on Forbes’ List of China’s Most Innovative Companies.^2^ The ByteDance’s quick growth and huge achievements are regarded as a disruption in the Internet industry. Moreover, this study mapped its disruptive innovation process using the key events trajectory method (Pettigrew, 1990). From a start-up to a leading firm, the whole process can be divided into three phases: centralized exploration, reshaping expansion, and self-drive reinforcement phase. The descriptions of the three phases are shown in Table 1. The analysis for each phase is detailed in the following sections.

**Table 1:** Description and division of the case company’s disruptive innovation process.

Timeline	Phase I: 2012–2014	Phase II: 2015–2019	Phase III: 2020-now
Phase division	Centralized exploration phase	Reshaping expansion phase	Self-drive reinforcement phase
Key disruptive innovation strategies	Launch and rise of the inferior but attractive product, Toutiao Toutiao’s transformation toward the content creation platform	Obtaining big success in the short video field Starting moving toward the foreign markets Operating multiple product lines in parallel, Establishing the development model of APP Factory	Formation of the core business matrix of “information distribution + content community + short video + overseas” Implementation of the Business Unit system and sorting out its business segment Comprehensive overseas strategic layout with products and services covering 150 countries and regions in 75 languages
Summary of period results	Identifying the user pain point of information overload when entering the mobile Internet era. Taking intelligent information distribution and recommendation as the entry point and introducing the AI technology quickly. Disrupting the traditional media industry pattern by building the recommendation engine.	Analyzing the development trend of short video field accurately and timely entering its market. Creating a series of short video APP products represented by Douyin and occupying the market quickly and roundly. Incubating multiple digital product lines and achieving rapid expansion through product diversification and globalization.	Establishing a comprehensive information platform and short video social platform as the core of the product matrix. Reorganizing the business units and focusing on the development of cross-border, multi-scene content platforms. Continuing the strategic layout of globalization

### Centralized Exploration Phase

#### Condition

In the initial period, the unsolved user demand and matured digital technology are the main conditions for ByteDance establishing its new information distribution pattern. In 2010s, the explosive growth of smartphones promoted the rapid increase of mobile information content supply. However, the information distribution pattern was still unified edition and undifferentiated distribution. This made it difficult for users to quickly find their interested contents in mass information. The problem of information overload was prominent. Meanwhile, the digital technologies such as 4G and big data gradually matured. Hence, ByteDance tried to utilize the emerging digital technologies to create a new pattern of intelligent information distribution, to meet the users’ personalized needs. As a start-up, ByteDance targeting at the information distribution market and building innovative pattern did not attract the incumbents’ attention. The coding results for this phase are shown in Supplementary Table A.

#### Action

##### First-Order Dynamic Capabilities Deployment

During the centralized exploration phase of disruptive innovation, ByteDance deployed its first-order dynamic capabilities (DCs). The three dimensions involve sensing potential digital entrepreneurship opportunities, seizing and leveraging external resources, and repositioning and refocusing the business.

ByteDance sensed the potential digital entrepreneurial opportunity from the contradictions between the traditional information distribution pattern and the users’ personalized needs. Consequently, it launched its first digital product, Toutiao. Toutiao is a mobile application that can realize the personalized push of information by utilizing artificial intelligence (AI) technologies and integrating the existed information. The earliest version of Toutiao highly relied on the external information sources. In the early promotion stage of Toutiao, ByteDance acquired customer resources and channel resources by establishing external cooperation. Hence, ByteDance’s seizing capability during the exploration period has focused on the full use of external resources.

Since the intelligent information distribution pattern highly relied on the external information source, the operational risks such as content infringement grew with its popularity. Thus, ByteDance began to transform into a content creation platform in 2014. This is an important strategic choice for ByteDance. The accumulated user base, technological advantages, and business exploration experiences served as the foundations for its transformation. Hence, in this phase, ByteDance also restructured its resources to establish the sustainable competitive advantages.

##### Focused Business Model Innovation

ByteDance’s business model innovation (BMI) in this phase was manifested as differentiated value proposition, exploitative value creation, and functional value capture. For the value proposition, ByteDance chose the information distribution market as the entry point, avoiding direct competitions with the giants. Then ByteDance combined the potential opportunities and itself technical advantages. Accordingly, ByteDance formulated its unique value proposition, using big data and AI recommendation technology to provide personalized information push.

In the value creation aspect, ByteDance carried out related activities mainly based on the external resources. For instance, in the early period, Toutiao only integrated the existed information. It owned no editor, without exporting new content. In its promotion stage, Toutiao adopted the strategies of binding installation with new mobile phones. It also cooperated with some mature APPs to acquire users quickly. Moreover, the uses’ pre-existing data, such as browsing habits and browsing records, were analyzed by Toutiao’s intelligent algorithms. Then, it can grasp users’ preferences to match with their interested information.

In terms of value capture, Toutiao explored its cashing channels based on the function of intelligent information distribution. For example, ByteDance used its AI algorithms to deliver advertisements intelligently. Due to the better marketing effects and lower costs, the advertising business has created considerable benefits for ByteDance in its early stage. Similarly, Toutiao also used its efficient traffic^3^ distribution mechanism to guide users or information for other products accurately. According to Foss and Saebi (2018), ByteDance’s business model innovation in the centralized exploration phase can be summarized as a focused BMI.

##### Interactions Between DCs and BMI

In the centralized exploration phase, there exist interactions between the dimensions of ByteDance’s first-order dynamic capabilities and its focused business model innovation. Firstly, the first-order dynamic capabilities had obvious driving effects on the focused business model innovation. Its sensed potential digital entrepreneurial opportunities directly impacted the proposed value proposition. With an integration of external information resources, user resources, and channel resources, ByteDance realized the value creation of personalized information push. Furthermore, its reconfiguring capability promoted the subsequent readjustments of its own business.

Secondly, ByteDance’s business model innovation activities promoted the evolution of its dynamic capabilities, to certain extent. The success of the intelligent information distribution pattern made it aware of opportunities in content creation. The exploitative value creation process made the direction of resource integration clearer and improved the accuracy of seizing capability. The initial benefits obtained in the value capture section further supported its transformation for content creation.

To sum up, in this phase, the first-order DCs of ByteDance have formed a strong driving force for its BMI. Conversely, the focused business model innovation played a certain role in promoting the evolution of its dynamic capabilities.

#### Result

**Figure 1:**
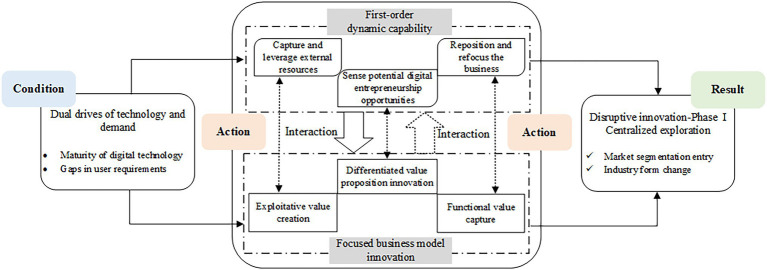
The happening mechanism of ByteDance’s centralized exploration phase.

Through the centralized exploration, ByteDance cut into the market segment of information distribution and created Toutiao, an intelligent information recommendation APP. Unlike with the incumbents in this field, the earliest version of Toutiao cannot produce any original news or contents, and its authority and accuracy cannot be ensured. Despite the inferior product, the intelligent information distribution is attractive to many users with which could help them solve the information overload problem. Consequently, as an Internet start-up, ByteDance obtained success by linking the market segment and emerging digital technology. Further, it completely changed the traditional pattern of batch aggregation, simple classification, and mechanical distribution. ByteDance’s centralized exploration in the early stages of disruptive innovation has allowed it to enter the news information field smoothly and gain a market position quickly. The happening mechanism of this phase is depicted in Figure 1.

### Reshaping Expansion Phase

#### Condition

In the second phase, the suitable development environment and new market opportunity constitute the main conditions for ByteDance’s further expansion. After an extensive market penetration, the smartphone’s performance and functions have been greatly improved. China’s communication operators also lowered the charge standard for 4G services. These conditions reduced the difficulty and costs of creating videos. Thus, some companies started to develop mobile applications for video creation, while the short video field is still in the early exploration stage. Meanwhile, the gradual saturation of smartphone market has caused the decline of China’s mobile Internet traffic dividend. The global Internet demographic dividend is shifting to Southeast Asia, South Asia, and South America. ByteDance needed to find new traffic channels to maintain its business growth. Hence, ByteDance considered the short videos as its next strategic attack direction and began trying to enter the overseas markets. The coding results for this phase are shown in Supplementary Table B.

#### Action

##### Second-Order Dynamic Capabilities Deployment

In the reshaping expansion phase of disruptive innovation, ByteDance deployed its second-order dynamic capabilities. The three dimensions involve sensing external and internal opportunities, seizing external and internal resources, and adjusting assets distribution flexibly.

First of all, ByteDance fixed on scanning the external environment and cultivating its perception ability. When the Internet giants did not realize the development prospects of short video field, ByteDance keenly identified this new opportunity. In the talent recruitment aspect, ByteDance emphasized learning ability to enhance the organizational perception ability.

Moreover, ByteDance paid attention to the full integration and utilization of internal and external resources. When entering the short video field, ByteDance quickly clarified its unique product positioning by analyzing the competitors and existing short video applications. During this period, ByteDance also launched TopBuzz, the overseas version of Toutiao, based on itself product operation experience.

In addition, ByteDance flexibly reconfigured its accumulated resources. To seize the first-mover advantage, ByteDance launched three short video applications for different user groups in 2016. It also migrated Toutiao’s recommendation algorithms and models into its short video products to ensure the distribution efficiency of videos. Overseas, its reconfiguring capability was reflected in conducting adaptive product innovation by embedding the local contexts.

##### Efficient and Complex Business Model Innovation

ByteDance’s business model innovation in this phase was manifested as inclusive value proposition, efficient value creation, and diversified value capture. For the inclusive value proposition, it determined by ByteDance’s expansion in multiple parallel product lines. In the short video field, ByteDance launched three distinct applications for different user groups. Meanwhile, ByteDance involved in multiple fields such as education, automobile, office, and launched related products, respectively. As for the overseas markets, ByteDance also launched multiple products to adapt the local markets, such as TikTok^4^ and Helo.^5^

For the value creation, related activities emphasized the efficiency from various aspects. In the product development aspect, ByteDance implemented agile development and rapid update pattern. This pattern realized continuous product optimization and rapid improvement of user experience. In the technical aspect, ByteDance established an artificial intelligence laboratory in 2016. This laboratory provided a solid foundation for the rapid iterative upgrade of subsequent products. In the organizational model aspect, ByteDance built an innovative system. This system was equipped with strong middle-end platforms as the supporting pillars, and lightweight front-end for rapid trials. As for the marketing aspect, ByteDance adopted a refined promotion and operation strategy to quickly attract the target customer groups. In addition, the content creation platforms represented by short video APPs have facilitated the deep resource links among multi-actors and promoted their value co-creation. These efficient value creation activities have jointly supported the rapid expansion of ByteDance.

In terms of value capture, the diversified product system strengthens the original value acquisition channels and creates new income sources. The short video products have further strengthened ByteDance’s advertising business with richer advertising forms and higher conversion rates. ByteDance also developed the e-commerce delivery and live broadcast services as new revenue sources. Within the ByteDance’s content production system, more frequent and complex value flows were promoted between content creators, consumers, advertisers, and the platform. In addition, the global market share of ByteDance has also increased with its products penetration toward overseas. According to Foss and Saebi (2018), ByteDance’s business model innovation in the reshaping expansion phase can be summarized as efficient and complex BMI.

##### Interactions Between DCs and BMI

In the reshaping expansion phase, there exist interactions between the dimensions of ByteDance’s second-order dynamic capabilities and its efficient and complex business model innovation.

Firstly, the second-order dynamic capabilities had an obvious driving effect on the efficient and complex business model innovation. The agile perception of new market opportunities has enabled ByteDance’s quick strategic deployments in promising areas, such as the short video field. The accurate judgments on its information distribution business’s development direction pushed its expansion forward the overseas market. In developing new products, ByteDance benefited from absorbing the external resources, and applying its existing intelligent distribution algorithms. Hence, the precise integration of the internal and external resources has led to efficient value creation and quick occupation of new markets. Also, the flexible adjustment on its resource allocation is essential when ByteDance migrated from the familiar information distribution market to other new fields. The strategy has ByteDance helped achieve scale expansion as a start-up, and opened more profit channels.

Secondly, the efficient and complex business model innovation has constantly stimulated the dynamic capabilities’ evolution. The inclusive value proposition innovation has forced ByteDance to maintain agile opportunity perception ability. The underlying entrepreneurial opportunities in diverse fields thus were identified. A series of efficient value creation activities has proposed higher requirements for the breadth, depth, and precision of resource acquisition. This has driven ByteDance to enhance its seizing capability. In establishing value capture channels, the demand for new revenue sources has inspired ByteDance to adjust and optimize the company’s resource allocation continuously. Consequently, its business layout has been restructured.

To sum up, ByteDance’s second-order DCs and efficient and complex BMI were driven mutually in this phase, with equally strong driving effects. Their frequent and interdependent interactions lead to the dynamic co-evolution of them.

#### Result

**Figure 2:**
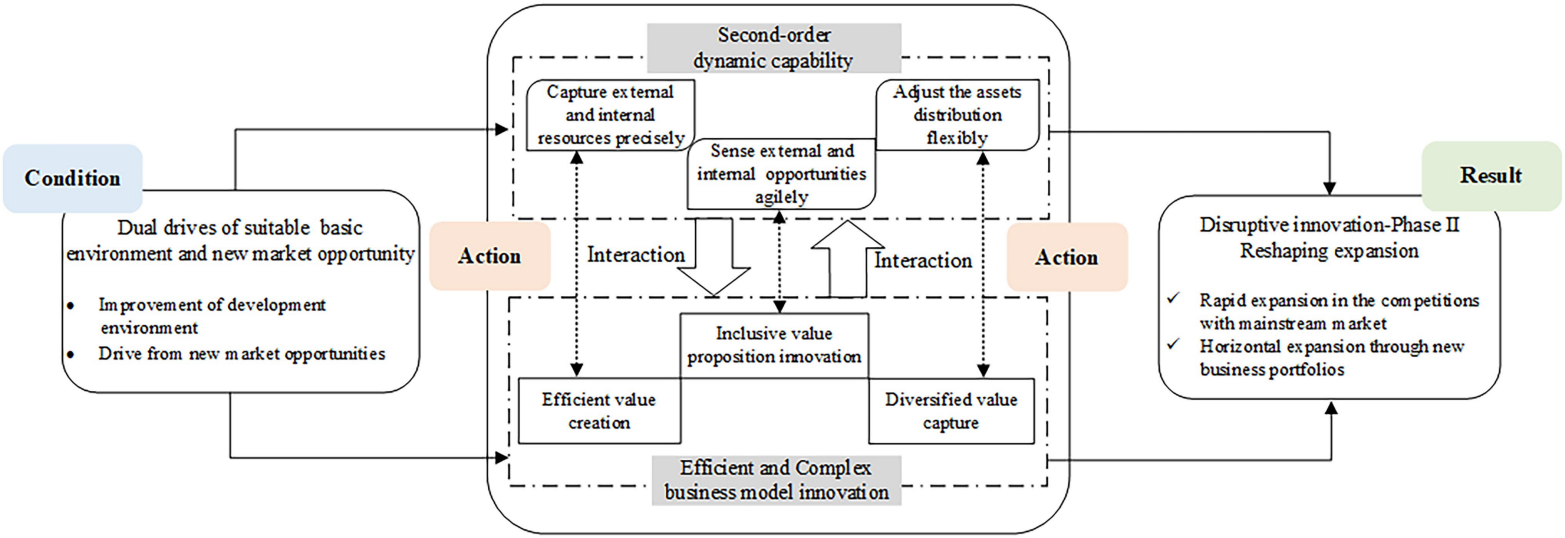
The happening mechanism of ByteDance’s reshaping expansion phase.

In this phase, ByteDance conducted vertical expansion to strengthen its mainstream market within and horizontal expansion to shape its new business portfolios. Through the reshaping expansion, ByteDance has grew into a direct competitor of the incumbents by taking market share from the giants. Unlike its unimpressive status in the first phase, ByteDance has become a company that cannot be ignored in the mainstream market of the Internet industry. Meanwhile, ByteDance has migrated its technological advantages from the information distribution field to several new markets represented by short video. With continuous investment in technology R&D, adherence to product innovation, and user-centered orientation, a batch of digital application products represented by Douyin were incubated. These products in new markets have performed excellently in terms of user size, user stickiness, and profitability. ByteDance’s reshaping expansion phase has served as a key step for its fulfilling disruptive innovation to achieve catching-up with the incumbents. The happening mechanism of this phase is depicted in Figure 2.

### Self-Drive Reinforcement Phase

#### Condition

The third phase, self-drive reinforcement, is also the current stage of ByteDance’s disruptive innovation. In this phase, the dual drives of active and passive self-disruption are the main conditions, caused by the increased uncertainties of external environment and emerged business bottlenecks. With the numerous subsequent imitators, intelligent information distribution tended to be saturated, and the competitive landscape has been relatively stable. Meanwhile, the sanctions on ByteDance’s products in foreign markets made its development prospects abroad seem very bleak. However, the sudden outbreak of COVID-19 in 2020 created a mass demand for online scenarios, such as games, information, and videos. These online needs became urgent under the physical isolation. Facing multiple obstacles and pressures from the main business, and underlying opportunities in the uncertain environment, ByteDance must implement disruption to itself actively and passively. Hence, ByteDance has moved forward a self-drive reinforcement phase of disruptive innovation. The coding results for this phase are shown in Supplementary Table C.

#### Action

##### Third-Order Dynamic Capabilities Deployment

In the self-drive reinforcement phase of disruptive innovation, ByteDance has deployed its third-order dynamic capabilities. The three dimensions involve hunting for all possible innovation opportunities, reconciling heterogeneous resources, and upgrading core competitiveness.

In this phase, ByteDance has established its product matrix, relatively complete core technology system, and management process. However, ByteDance refuses to set limits and hopes to explore greater opportunities for creativity and innovation. Accordingly, ByteDance has focused on the exploratory integration of heterogeneous resources at this phase. For instance, in 2020, the public’s demand for New Year movies during the Spring Festival cannot be satisfied due to the epidemic. ByteDance, never involved in the film and television industry, quickly bought the exclusive broadcast right of popular movies on the Internet. This cross-border resource integration behavior has largely promoted great reputation and business growth.

In addition, ByteDance has restructured its business layout and tried to upgrade its competitive advantages through cross-border expansion and self-optimization. On the one hand, ByteDance has applied the established traffic and technical advantages to the investments in multi-scenario content platforms. On the other hand, ByteDance is gradually building the search business based on its recommendation algorithms. As a result, an integrated and ecological closed loop of information connection is being formed. It is an optimization for its own business system which can build competition barriers to prevent being disrupted.

##### Evolutionary Business Model Innovation

ByteDance’s business model innovation in this phase was reflected in enhanced value proposition, ecological value creation, and cross-boundary value capture. For the value proposition, ByteDance has enhanced its value proposition around the corporate vision of “Global Creation and Exchange Platform.” Hence, ByteDance is committed to becoming a globalized and platform-based enterprise.

In the value creation aspect, relevant activities reflect obvious ecological characteristics. ByteDance has established its unique value creation logic, using the recommendation algorithms as tools and the content platforms as carriers. ByteDance is trying to integrate this logic into other unfamiliar but potential fields, such as online education, and finance, to incubate more innovative digital products. This cross-border innovation strategy aims to form a more robust, self-growing product ecosystem. Moreover, ByteDance has also implemented the business unit system for its business lines in 2021. The adaptative adjustment for its organizational structure also facilitates the construction and evolution of the platform ecosystem.

In terms of value capture, ByteDance has focused on reinforcing its sustainable competitive advantages. Besides strengthening its powerful businesses, ByteDance has also made extensive layouts in new fields such as games and enterprise services to establish multi-dimensional profiting channels. In addition, ByteDance has tried to expand the coverage of its products and services by advancing its globalization strategy to obtain more traffic and broaden the cashing avenues. According to Foss and Saebi (2018), ByteDance’s business model innovation in the self-drive reinforcement phase can be summarized as an evolutionary BMI.

##### Interactions Between DCs and BMI

In the self-driven reinforcement phase, there exist interactions between the dimensions of ByteDance’s third-order dynamic capabilities and its evolutionary business model innovation. Firstly, the three dimensions of third-order dynamic capabilities still play a strong supporting role in the three elements of the evolutionary business model innovation. This is consistent with the first two stages. Secondly, it is noteworthy that ByteDance’s business model innovation in this phase has shown a more significant role in promoting its dynamic capabilities.

In particular, the corporate vision of platformization and globalization has driven its development of sensing capability to hunt for all possible innovation opportunities. Furthermore, ByteDance strives to widely apply its competitive value creation logic to related scenarios, to form a self-organizing and self-evolving ecosystem. This process has directed its seizing capabilities to acquire and reconcile heterogeneous resources. Finally, the multi-level and multi-dimensional value capture mechanism built by cross-border innovation, has given more space for ByteDance’s transforming and reconfiguring capabilities.

To sum up, in this phase, the third-order DCs of ByteDance have supported the evolution of its BMI. Conversely, the evolutionary business model innovation has strongly promoted the cultivation of dynamic capabilities.

#### Result

**Figure 3:**
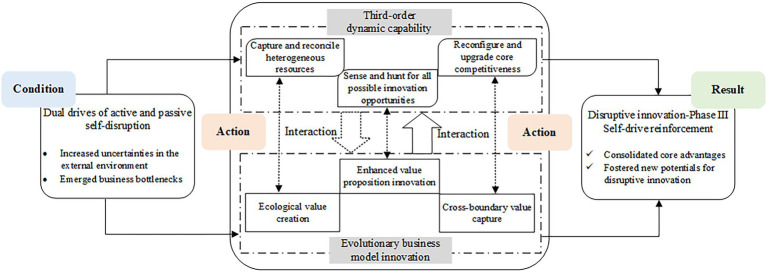
The happening mechanism of ByteDance’s self-drive reinforcement phase.

In the self-driven reinforcement phase, ByteDance has achieved an evolutionary growth in its existed and new businesses. Particularly, ByteDance has consolidated its position in the mainstream market by strengthening its core advantages. For example, ByteDance is still in the first echelon in the short video industry and maintains a stable gap with its competitors. Meanwhile, ByteDance has strived to incubate new disruptive innovation potentials. Its business matrix is being expanded and enriched based on the existed information distribution, content community, short video, and overseas layout. The reinforcements of the existing advantages and the cultivation of new growth points have jointly promoted the disruptions for ByteDance itself and the evolution of its innovation ecosystem. The happening mechanism of this phase is depicted in Figure 3.

## Discussion

### The Evolution Mechanism of Disruptive Innovation for Start-Ups in the Digital Era

**Figure 4:**
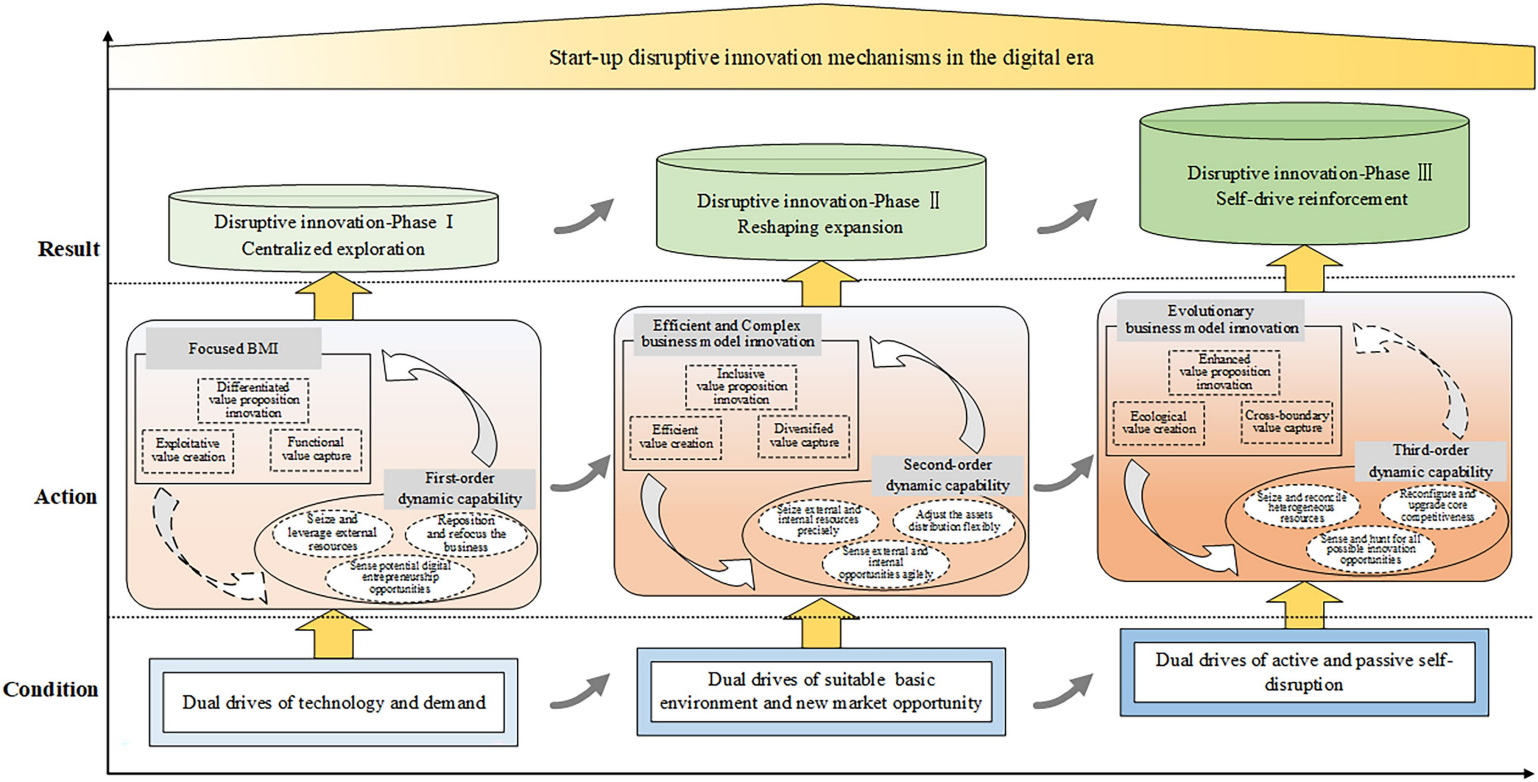
The evolution mechanism of start-up disruptive innovation in the digital era.

According to the case analysis, this study proposes the evolution mechanism model of start-up disruptive innovation in the digital era, as displayed in Figure 4. The evolutionary process of start-up digital disruptive innovation has experienced three phases successively: centralized exploration, reshaping expansion, and self-drive reinforcement.

Considering each phase from the longitudinal axis, the happening mechanism follows the logic of condition-action-result. For the centralized exploration phase, it originates from the dual drives of digital technology progress and users’ underlying demand. Accordingly, the core actions, dynamic capabilities, and business model innovation, are developed into the first-order DCs and focused BMI. In this phase, both focused on the external resources and opportunities. For their interactions, the first-order dynamic capabilities strongly guide and support the focused business model innovation. The focused business model innovation slightly facilitates the evolution of dynamic capabilities toward higher order. Consequently, the start-up can establish differentiated competitive advantages and obtain the initial niche in the targeted market segmentation by responding to the sensed opportunities.

For the reshaping expansion phase, it starts due to the improved technology and policy conditions as well as new market opportunities. In this phase, the second-order DCs and efficient and complex BMI highlights leveraging both external and internal resources and opportunities. Meanwhile, the two categories of actions keep dynamic alignments through their strong and iterative interactions, leading to their co-evolutions. The process effectively accelerates the penetration into the mainstream market and supports the rapid expansion toward multiple new business fields. Consequently, the start-up grows into a competitor of the existing incumbents which cannot be ignored, and changes the competitive landscape.

For the self-drive reinforcement phase, it aims to maintain and enhance the achievements of disruptive innovation. Since business disruption always occurs uncertainly and unpredictably, the drive forces in this phase mainly come from the pressures of active and passive self-disruptions. Accordingly, the third-order DCs and evolutionary BMI concern on reconfiguring the owned resources and developing its ecosystem for open innovation and cross-boundary innovation. For their interactions, the transition of third-order dynamic capabilities still supports the implementation of BMI. Meanwhile, the pull of BMI for the dynamic capabilities’ evolution is obviously strengthened by posting new requirements for different capabilities. Consequently, as a mainstream enterprise, it strives to reinforce its core advantages and nurture new disruptive innovation potentials. The goal is to form the competitive barriers and prevent being disrupted.

Considering the three phases from the horizontal axis, their evolution process conforms to the characteristics of disruptive innovation. From centralized exploration in the non-mainstream market to expansion toward the mainstream market, and reinforcing the new industry landscape. The three phases evolve dynamically to realize disruptive innovation. In parallel, the two key actions of dynamic capabilities deployment and business model innovation also experience through continuous evolutions.

For the dynamic capabilities, sensing, seizing, and reconfiguring constitute the main dimensions of the first-, second-, and third-order DCs. The lower-order DCs in earlier phase serve as the foundations for the higher-order ones in the subsequent phase. For the business model innovation, the process is presented as from focused BMI, to efficient and complex BMI, and to evolutionary BMI. Its key elements, value proposition, value creation, and value capture, are extended and adapted continuously. The interactions between the DCs and BMI facilitate their dynamic alignments in each phase, also the transition of DCs and the evolution of BMI across phases. This is also the essential mechanism underpinning the advancement of the entire disruptive innovation trajectory.

### The Fulfillment Path of Start-Up Disruptive Innovation in the Digital Era

**Figure 5:**
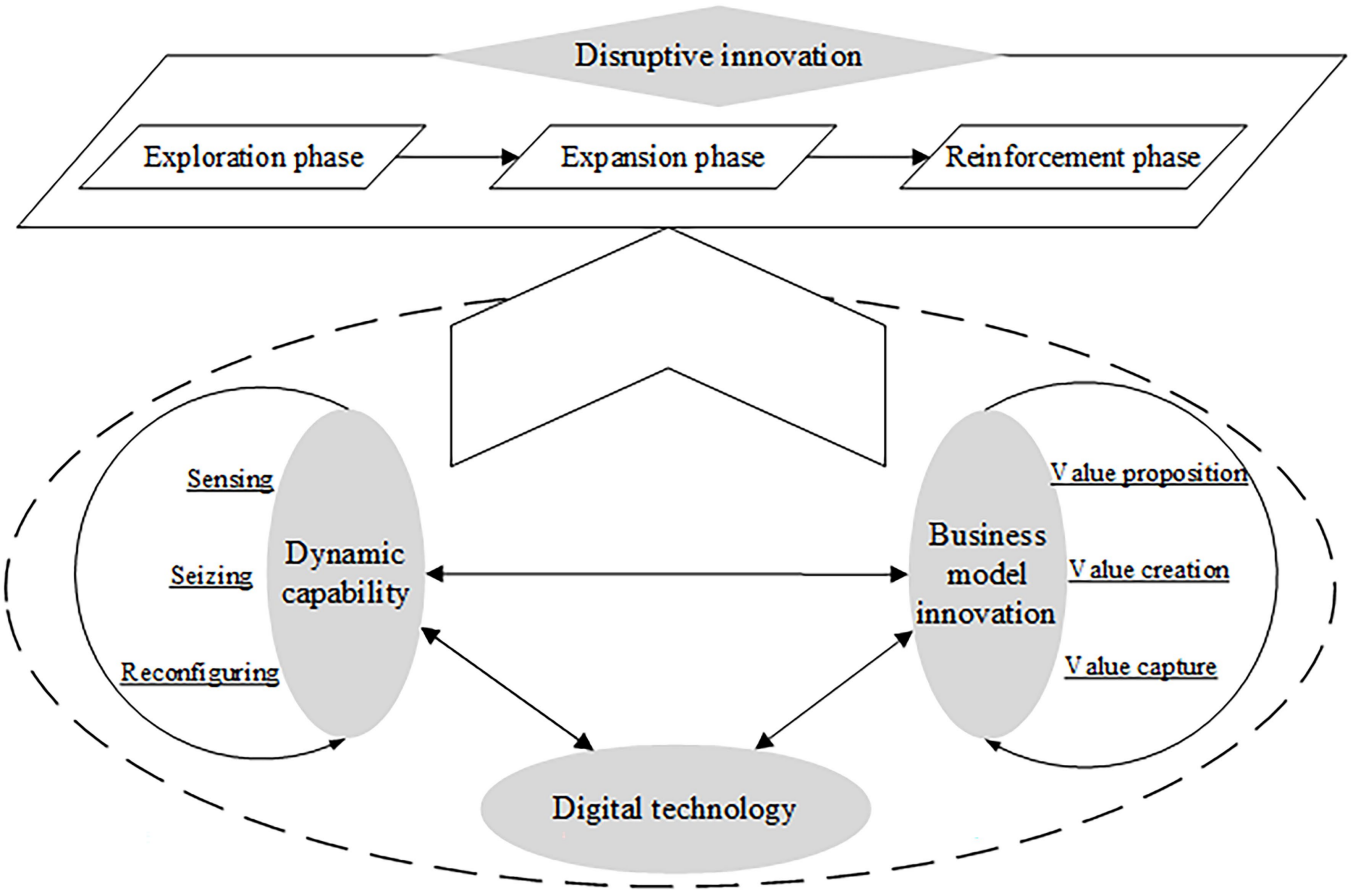
The conceptual model of start-up disruptive innovation path in the digital era.

Although the unique characteristics of each phase in the disruptive innovation process, repeated patterns over the three phases were observed and can be generalized. Therefore, this study inductively proposes a conceptual path model of start-up disruptive innovation in the digital era in Figure 5. This model shows that the disruptive innovation process for start-up consists of three phases: exploration, expansion, and reinforcement. There are three key pillars in this process: digital technology, dynamic capabilities, and business model innovation. They jointly support this evolutionary process by playing different roles.

Digital technology is an antecedent and an element for disruptive innovation in the digital era, which is indispensable. In particular, the emergence and infusion of digital technology create a dynamic and uncertain competitive environment, providing opportunities for a start-up to implement disruptive innovation. In each phase of disruptive innovation, digital technology participates in various activities by influencing the evolution of dynamic capabilities and business model innovations. Inversely, the evolutions of dynamic capabilities and business model innovation facilitate the progress of digital technology.

The dynamic capabilities and business model innovation can be regarded as the two core elements of disruptive innovation. Dynamic capabilities can be characterized as three dimensions, sensing, seizing, and reconfiguring, while business model innovation as value proposition, value creation, and value capture. Distinct dimensions of dynamic capabilities support and drive the innovation of different elements in the business model. The business model innovation activities constantly promote the transition of dynamic capabilities toward a higher order. Hence, the interactions between them realize their alignments and co-evolutions, constituting the core mechanisms for disruptive innovation.

Based on the analysis above, this study proposes the following propositions.

*Proposition 1*. The start-up disruptive innovation in the digital era is a dynamic and evolutionary process. Its path can be developed through three phases: exploration, expansion, and reinforcement.

*Proposition 2*. There are three key pillars for start-up to fulfill disruptive innovation in the digital era, digital technology, dynamic capabilities, and business model innovation. Their dynamic interactions jointly support this process.

*Proposition 2a*. Digital technology is the basic infrastructure with multiple roles through the disruptive innovation process. It is the induction condition for digital disruptive innovation and part of the results.

*Proposition 2b*. Dynamic capabilities and business model innovation are essential actions for start-ups’ disruptive innovation in the digital era. The constant interactions between them facilitate their co-evolutions and constitute the core mechanisms of disruptive innovation in the digital era.

## Conclusion

### Theoretical Contributions

The findings from this study make several contributions to the current literature. Firstly, this study investigates the star-up disruptive innovation process embedded in the digital context. The emergence and rapid infusion of digital technologies are enabling digital entrepreneurship, which further triggers the potential of disruptive innovation. Despite the related business practices, the academics still lack sufficient theoretical explanations. By investigating a Chinese Internet start-up’s digital entrepreneurship process, which achieves disruptive innovation, this study responds to the call concerning the new context of disruptive innovation (Si and Chen, 2020). Moreover, this study is also the initial effort to establish the link between disruptive innovation and digital entrepreneurship literature.

Secondly, this study contributes novel theoretical insights into the evolution mechanism and fulfillment path of start-up disruptive innovation in the digital era. The proposed evolution mechanism model unveils the start-up disruptive innovation trajectory in the digital era thoroughly, and explains its happening mechanism explicitly. The abstracted fulfillment path model provides a conceptual framework for identifying the three phases of start-up disruptive innovation in the digital era and key pillars. The two models have offered systematical understandings from a process view which are helpful to open the black box of start-up disruptive innovation in the digital era. Therefore, this study can be regarded as a dialogue bridge between the related practical phenomenon and academic literature.

Thirdly, this study consolidates the links between the business model innovation and dynamic capability literature. Based on the case analysis findings, the interactions between the dynamic capabilities and business model innovation are identified as the core action mechanism supporting the start-up disruptive innovation in the digital era. For each phase, the dynamic capability and business model innovation keep align through their interactions. For the evolution across phases, their interactions promote the advancement of disruptive innovation. Moreover, their interaction patterns distinguish in different phases. By providing an integrated perspective to deepen understandings of the fulfillment of disruptive innovation, this study extends and enhances the previous related studies (Franco et al., 2021) on their links.

### Managerial Implications

This study provides important implications for practitioners concerned with disruptive innovation in the digital context.

Firstly, those start-ups intending to implement disruptive innovation through digital entrepreneurship could systematically perceive the disruptive innovation from an evolutionary process view. The start-ups should realize the evolution logic of disruptive innovation to establish overall strategic planning. By knowing the characteristics of organizational behaviors in different phases, the start-ups could allocate their limited resources and attention to suitable places to improve their innovation efficiencies. In addition, the start-ups can also utilize the proposed evolution mechanism models as an examination tool to locate their status and provide guidance for the next actions.

Secondly, potential entrepreneurs are recommended to keep clear about the key components that support the fulfillment of disruptive innovation. Furthermore, an in-depth understanding of their connotation and unique roles in disruptive innovation is required. For the digital technologies or infrastructures, potential entrepreneurs should fully leverage their disruptive potentials while balancing the investment for them so as to avoid the digitalization paradox. For the dynamic capabilities, entrepreneurs need to utilize and integrate the capabilities in different dimensions flexibly. For business model innovation, the choice for the specific business model innovation type is necessary to fit with the development phase.

Thirdly, managers require developing an agile mindset in their disruptive innovation practices to nurture organizational flexibility, especially in the digital era. With the turbulent environment and increasingly ambiguous organizational and industry boundaries, disruptors can also be disrupted. One of the available means to solve this is to emphasize the interactions between dynamic capabilities and business model innovation. Although the start-ups own the inherent organizational agility, the leaders still need to keep cautious about the misalignments between the organizational dynamic capabilities and business model and try to facilitate their co-evolution to sustain the disruptive innovation advantages.

### Limitations

The limitations of this study should be acknowledged. Firstly, the research findings were drawn from the limited evidence of a Chinese Internet company. The limitation of single case study can be improved by using multiple case studies to enhance the findings’ reliability. Considering the different industry or country contexts, future research can supply cases from other industries or economic entities to derive more fine-grained conclusions. Secondly, this study investigates the case company’s disruptive innovation process based on the data collected by the end of 2021. It is recommended to keep tracking the case and infuse other theoretical perspectives to obtain renewed understandings. Finally, the proposed conceptual model initially explores the logic links among the three key constructs of digital technology, dynamic capabilities, and business model innovation. Using other empirical paradigms such as hypothesis testing (Khan et al., 2022) or qualitative comparative analysis (QCA) to examine their relationships quantitatively is also a fruitful inquiry line.

## Data Availability Statement

The raw data supporting the conclusions of this article will be made available by the authors, without undue reservation.

## Author Contributions

KZ contributed to draft writing, case analysis, and basic text preparation. LF and JW contributed to concept preparation and supervisory work. GQ helped in data collection and case analysis. HL contributed to case analysis and draft revision. All authors contributed to the article and approved the submitted version.

## Funding

This research was funded by Innovation Method Fund of China with grant number 2018IM020300, 2019IM020200; Joint Funds of the National Natural Science Foundation of China (Project No. U1904210-4); Zhengzhou University Support Program Project for Young Talents and Enterprise Cooperative Innovation Team (Project No. 132-32320423); Shanghai Science and Technology Program (Project No. 20040501300); and General Project of Humanities and Social Science Research for Henan Province’s Colleges and Universities (Project No. 2023-ZZJH-039).

## Conflict of Interest

The authors declare that the research was conducted in the absence of any commercial or financial relationships that could be construed as a potential conflict of interest.

## Publisher’s Note

All claims expressed in this article are solely those of the authors and do not necessarily represent those of their affiliated organizations, or those of the publisher, the editors and the reviewers. Any product that may be evaluated in this article, or claim that may be made by its manufacturer, is not guaranteed or endorsed by the publisher.

## Footnotes

^1^ www.cbinsights.com

^2^ www.forbeschina.com

^3^The meaning of traffic in this study: the activity of data and messages passing through an online communication system or the number of visits to a particular website (source: https://dictionary.cambridge.org/).

^4^The international and oversea version of Douyin, launched for the global market.

^5^A local-language social media platform, firstly launched for the Indian market.
