# Supplementary material for: Start-Up’s Road to Disruptive Innovation in the Digital Era: The Interplay Between Dynamic Capabilities and Business Model Innovation
Source: Front Psychol. 2022 Jun 21;13:925277. doi: 10.3389/fpsyg.2022.925277 (PMC9255633; doi:10.3389/fpsyg.2022.925277)
Supplement: Supplementary file 1 [file Data_Sheet_1.docx]

Supplementary Material

# Supplementary Tables

Table A The coding results of ByteDance disruptive innovation in the exploration phase

| Code type | Core category | Main category | Subcategory | Evidence |
| --- | --- | --- | --- | --- |
| Condition | Dual drives of technology and demand | Maturity of digital technology | Digital technology development brings opportunities | Smartphones grew explosively, and the 4G communication technology was increasingly matured. |
|  |  | Gaps in user requirements | Information overload becomes a major user pain point | There was a lot of mobile news content, making it difficult for users to find their interesting content quickly. |
| Action | First-order dynamic capability | Sense potential digital entrepreneurship opportunities | Linking user pain points with technology development | We analyzed the mismatch between existing product and service supply and the target customers' needs. We then found tools to solve the problem, i.e., big data and AI technology. |
|  |  | Seize and leverage external resources | Crawling existing information resources | At first, Toutiao was just a carrier of various website contents. |
|  |  |  | Introducing initial user resources | When the user logs in for the first time with the account of another APP, the algorithm can interpret their interests within 5 seconds and push the content that the user wants to watch. |
|  |  |  | Integrating channel resources | It expanded the promotion channels by cooperating with mobile phone manufacturers. |
|  |  | Reposition and refocus the business | Moving from pure content integration to content creation | Toutiao began to encourage users to contribute their original content, to reduce the operational risks of content infringement. |
|  | Focused business model innovation | Differentiated value proposition | Innovative value proposition based on user orientation and technology empowerment | We decided to use big data and AI recommendation technology to provide personalized information push.  Algorithms have no values. What is good for the user is good  The slogan is "What you care about is the headline (Toutiao)". |
|  |  | Exploitative value creation | Value creation activities based on the integration of external resources | ByteDance did not export new content in the early development stage.  Toutiao provided the binding login entry for other mature social networking APPs.  We paid close attention to users' feedback and used the algorithm to update the user model in real time to achieve high customization.  Adopting a mobile phone pre-installation strategy to occupy channels |
|  |  | Functional value capture | Building precise advertisement marketing model and traffic sharing model | The AI algorithms are used to intelligently and accurately place advertisements to realize cashing.  Toutiao can guide the target users or messages for other product partners through the traffic distribution technology. |
|  |  |  | Opening up revenue channels by its own characteristics | In 2014, "Toutiaohao", a self-media platform, was launched to support content entrepreneurship and pushed out a premium paid membership system. |
| Result | Centralized exploration | Market segmentation entry | Becoming a leader in the news information industry | Toutiao's advantages in the news and information field continue to expand, becoming the leader in this field. |
|  |  | Industry form change | Breaking the traditional information distribution model | Toutiao has completely changed the previous media batch summary, simple classification, mechanical distribution model |

Table B The coding results of ByteDance disruptive innovation in the expansion phase

| Code type | Core category | Main category | | Subcategory | | | Evidence |  |
| --- | --- | --- | --- | --- | --- | --- | --- | --- |
| Condition | Dual drives of suitable basic environment and new market opportunity | | Improved development environment | | Improvement of hardware function  Perfection of video technology | The performance of smartphone hardware (cameras, processors, etc.) continued to be improved. And the AI video functions were enriched, which greatly reduced the threshold of short video creation. | | |
|  |  | |  | | Friendly policy environment | In 2015, China's telecommunications operators lowered the charge standards for 4G packages. | | |
|  |  | | New market opportunities | | Industry survival pressure | The Internet traffic dividend has almost peaked. ByteDance urgently needed new traffic channels to maintain the business growth.  The global Internet demographic dividend has shifted from China to Southeast Asia, South Asia, and South America. | | |
|  |  | |  | | Emergence of short video niche | In 2015, ByteDance planned the short video as its next strategic direction. This field is still in the early stage of exploration. | | |
| Action | Second-order dynamic capability | | Sense external and internal opportunities agilely | | Being keenly aware of external market opportunities | When the growth inflection point of the short video field came, the giants did not pay enough attention to developing related short video applications. | | |
|  |  | |  | | Emphasizing the development of organizational perception capabilities | ByteDance believes fit is the most important, and seeks adults with learning ability, broad vision, and mature minds. | | |
|  |  | | Seize external and internal resources precisely | | Assimilating external resources accurately | In 2016, Facebook hesitated to acquire the well-known short-video APP, Musically. ByteDance forestalled to take control of the company. ByteDance also acquired Flipagramm, an American short video APP, and invested in several overseas products. | | |
|  |  | |  | | Competitor analysis  Product design positioning | "We collected almost all the short video applications to see how they interact. Then we reflected on what kind of technology should be used to realize it and make something unique." | | |
|  |  | |  | | Flexible use of own experience | Toutiao's overseas business relied on its domestic marketing experiences and solid technical support. It also considered overseas consumption habits to develop localized products. | | |
|  |  | | Adjust assets distribution flexibly | | Launching multiple differentiated products in the same field | ByteDance intensively launched three short video applications to seize the industry trend. | | |
|  |  | |  | | Building its own platform to expand the business scope | Douyin broke through the technical bottleneck and built its own e-commerce platform. | | |
|  | Efficient and Complex business model innovation | | Inclusive value proposition | | Distinguishing and fully covering the customer demand | The three short video APPs launched by ByteDance, targeted at the users in different regions and ages. They adopted the differentiation competitive strategy to occupy the short video market maximumly. | | |
|  |  | | Efficient value creation | | Sticking to investing in technological innovation | ByteDance established an artificial intelligence lab in 2016. This lab is responsible for providing technical services for key products and participating in finding solutions.  ByteDance started the evolution of its basic infrastructure 2.0 in 2018. After integration, it provided three major infrastructures of storage, computing, and R&D systems. They can span offline and online and become a common base to support all product lines. | | |
|  |  | |  | | Agile product innovation | In the early stage, Douyin focused on improving the product functions and adopted the method of "agile development, small steps, and fast running". The new version was updated almost every ten days.  Douyin has adopted a new product interaction interface with cold start, full-screen, and automatic display. It has subverted the original short video APPs’ horizontal screen and one-screen display model. | | |
|  |  | |  | | Continuous improvement of user experience | Douyin has tried to identify and push higher-quality videos by improving the reporting and reviewing mechanism.  Douyin launched a variety of theme activities officially to encourage users to participate in the creation and interaction. The strategy has enriched the contents in various vertical fields. | | |
|  |  | |  | | Adaptive innovation of the organizational model | ByteDance has gradually built a system with strong middle-end platforms as the support, and lightweight front ends for quick trials. ByteDance has become a light APP factory driven by this system to support its business’s rapid iterations. | | |
|  |  | |  | | Matching suitable marketing strategies with the product characteristics | Douyin has actively developed a variety of promotion channels, including employing the Internet and entertainment celebrities, sponsoring popular variety shows, and advertising on the prevalent social platforms. They were the key boosts for its initial cold start. | | |
|  |  | |  | | Efficient integration of resources oriented by value co-creation | Whether the creators as supply side, or advertisers and users as the demand side, the short video APP has realized the deep links between the platform and its partners. Meanwhile, it also has a stronger incentive and guidance effect for ordinary users. | | |
|  |  | | Diversified value capture | | Strengthening the advertising business revenue | Douyin’s revenue mainly came from information push advertisements and open-screen advertisements.  The unit price of advertisements on short video platforms is significantly higher than on other types of platforms due to its high conversion rate. | | |
|  |  | |  | | Exploiting the e-commerce business income | Douyin e-commerce has charged different commissions according to different channels, such as self-operated Douyin stores, or linking with the third-party e-commerce platform.  In 2018, ByteDance launched the first independent e-commerce APP. | | |
|  |  | |  | | Forming the multi-actor benefiting mechanism | The short video platform represented by Douyin has established a cooperative and orderly content production system. It has promoted the generation of creativity and brought new opportunities for short video’s commerce. Further, the video producers, consumers, advertisers, and the platform can jointly share the benefits. | | |
|  |  | |  | | Initiating the overseas market layout | In 2015, TopBuzz, Toutiao’s overseas version, was officially launched. Then, a series of products, including TopBuzz Video (overseas version of watermelon video), TikTok (overseas version of Douyin), Vigo Video (overseas version of volcano video) were launched successively. In 2018, ByteDance launched Helo, a local-language social media platform for the Indian market. | | |
| Result | Reshaping expansion | | Rapid expansion in the mainstream market | | Taking the market share from the giants | ByteDance has become the second-largest online advertising platform after Alibaba. The proportion of ByteDance's products has ranked second only to Tencent, regarded as the APP series with the fastest growth in user time. | | |
|  |  | | Horizontal expansion through new business portfolios | | Opening and occupying the new market successfully | The number of users and the total monthly usage time in the short video industry have grown rapidly. The short video industry now is the second-largest industry in the mobile Internet. Douyin has become the first-echelon product in the short video industry. | | |

Table C The coding results of ByteDance disruptive innovation in the reinforcement phase

| Code type | Core category | Main category | Subcategory | Evidence |
| --- | --- | --- | --- | --- |
| Condition | Dual drives of active and passive self-disruption | Increased uncertainties in the external environment | Stimulus of external crisis | The emergence of COVID-19 has strengthened the application of online scenarios. New opportunities were then brought for the short video industry where the mobile Internet traffic dividend is fading. |
|  |  | Emerged business bottlenecks | Saturation of the main business market | The market of news and information distribution applications has been relatively mature. The traffic within this market has almost reached its upper limit. |
|  |  |  | Obstruction of globalization strategy | The sanctions on TikTok have been imposed by US and India. There is widespread concern in the market about ByteDance’s overseas development prospect. |
| Action | Third-order dynamic capability | Sense and hunt for all possible innovation opportunities | Eager for innovation and refuse to set limits | Yiming Zhang (Ex-CEO of ByteDance) said that “ByteDance was fortunate to seize the opportunity of the development of the times. But do not stop there, be more creative and meaningful”. |
|  |  | Seize and reconcile heterogeneous resources | Exploitative resource integration | In the Spring Festival of 2020, many movies had to be withdrawn due to COVID-19. ByteDance quickly bought the exclusive online broadcast rights of the popular movie *Lost in Russia* by 98.9 million dollars. People's demand for enjoying new year movie at home was satisfied. It created the first record of Internet online premiere for Spring Festival Movies. |
|  |  |  | Explorative resource integration | Toutiao has attracted nearly 300 million monthly active users since its launch. A large amount of content data accumulated by the users can be available information source for launching the search products. |
|  |  | Reconfigure and upgrade core competitiveness | Breaking through the industry pattern | With the acquired users and technical advantages, ByteDance is focusing its investments on the content industry and platform building. |
|  |  |  | Building competition barriers | ByteDance is building an ecological closed loop of information connection by integrating the algorithmic recommendation and user search. It aims to promote more efficient connections between users and information. |
|  | Evolutionary business model innovation | Enhanced value proposition | Focusing on the vision of globalization and platform enterprise | ByteDance will adhere to the corporate vision of "Global Creation and Communication Platform." |
|  |  | Ecological value creation | Construction of the product matrix | ByteDance has built a product matrix with two major platforms, the general information platform and the video social platform. ByteDance has used the recommendation algorithm as the tool and the content platform as the carrier. There are even several products in the same field. |
|  |  |  | Cross-border innovation based on the core business system | ByteDance has implemented diversified operations. Its business territory has been extended to finance, e-commerce, online education, social networking, and other fields. |
|  |  |  | Dynamic adjustment of organizational structure | ByteDance has adjusted its business lines into several business units in 2021. As a result, six business segments were established: Douyin (short video), Dali Education (online education), Lark (office suite), Volcano Engine (enterprise service), NUVERSE (game), and TikTok (overseas market). |
|  |  | Cross-boundary value capture | Enhancing sustainable competitive advantage | “We are trying to build a multi-type information distribution mechanism that cooperates with each other, to retain more traffic in our product circle.” |
|  |  |  | Developing multi-dimensional profit channels | Besides advertising, ByteDance has explored other cashing ways by actively deploying new fields, such as searching, games, and enterprise services. |
|  |  |  | Deepening globalization layout | ByteDance is making steady progress toward internationalization. Its products and services, led by the short-video app Tiktok, are now available in 75 languages in 150 countries and regions. And they have been at the top of the app store ranking list in more than 40 countries and regions. |
| Result | Self-drive reinforcement | Consolidated core advantages | Consolidating the mainstream market position | The short video industry has maintained a steady growth trend where ByteDance keeps in the first echelon. |
|  |  | Fostered new potentials for disruptive innovation | Forming core product system | ByteDance has built a core business matrix based on its information distribution, content community, short video, and overseas market. Its user stickiness continues to enhance, and the contents continue to enrich. |
